# Dataset on the Life Cycle Assessment of fructo- and galacto-oligosaccharides (FOS and GOS) produced by synthesis or hydrolysis

**DOI:** 10.1016/j.dib.2022.108478

**Published:** 2022-07-18

**Authors:** Esteban Gerbino, Camille Quentier, Caroline Pénicaud

**Affiliations:** a Center for Research and Development in Food Cryotechnology (CIDCA, CCT-CONICET La Plata), La Plata, Argentina; b Université Paris-Saclay, INRAE, AgroParisTech, UMR SayFood, 91120, Palaiseau, France

**Keywords:** Prebiotics, Innovative production processes, Life Cycle Assessment, Food engineering

## Abstract

The prebiotics like FOS and GOS are receiving special attention in the food industry due to their potential health benefits. They can be produced by enzymatic synthesis by using disaccharides or other substrates as raw materials or by extraction and hydrolysis from different natural sources (roots, legumes). However the environmental footprints of these different production schemes are lacking. This dataset presents Life Cycle Assessment (LCA) of the production of FOS and GOS by enzymatic synthesis from glucose (to get FOS) or lactose (to get GOS) and hydrolytic production from extraction of yacon potato (to get FOS) or chickpea (to get GOS). A cradle-to-gate approach was considered in the two scenarios under assessment (the phases of use and/or final disposal of FOS/GOS were not considered). The functional unit was defined as 100 g of FOS/GOS produced. LCAs were performed using data collected at the laboratory scale during experiments, supplemented with data from technical and scientific literature. Ecoinvent database provided background data. SimaPro was used for the LCA modeling with the midpoint impact EF2.0 characterization method to calculate environmental impacts. For each scenario (FOS produced by synthesis, FOS produced by hydrolysis, GOS produced by synthesis, GOS produced by hydrolysis), the Life Cycle Inventory (LCI) and the Life Cycle Impact Assessment (LCIA) are provided. These data can be used (i) to identify the main environmental hotspots of the production process, (ii) to compare the different process alternatives between them and (ii) to suggest eco-design options to upscale these processes. They could also be re-used in other LCA studies which would include FOS and/or GOS in the production system.

## Specifications Table

**Table:** 

Subject	Environmental Science
Specific subject area	Environmental assessment of the production of fructo- and galacto-oligosaccharides (FOS and GOS) produced by synthesis or hydrolysis
Type of data	Table
How the data were acquired	Inventory data were obtained either by manual measurements or by calculations, or found in the technical and scientific literature. Background data come from the database Ecoinvent 3.5. Life Cycle Assessments were computed by using SimaPro software (v9.0.0.35) and the EF Method (adapted) V2.00 / Global (2010)/with tox categories to obtain the midpoint indicators presented in this paper.
Data format	Raw and analyzed
Description of data collection	Inventory data were collected during experiments performed in 2018 in CIDCA-CONICET (La Plata, Argentina) for synthesis process and University of Madeira (Madeira, Portugal) for hydrolysis process. Manual measurements of data have been completed by collection of data from technical literature. Calculations have been performed to quantify energy flows. Scientific literature has also be used as a source of data.
Data source location	Experiments for synthesis process:Institution: CIDCA-CONICETCity/Town/Region: La PlataCountry: ArgentinaExperiments for hydrolysis process:Institution: University of MadeiraCity/Town/Region: MadeiraCountry: PortugalCompletion with literature data and LCA computing:Institution: INRAECity/Town/Region: Thiverval-GrignonCountry: France
Data accessibility	Repository name: Data INRAEDirect URL to data: 10.15454/BRBK8X

## Value of the Data

• This dataset presents a unique Life Cycle Inventory and Life Cycle Impact Assessment of the production of fructo- and galacto-oligosaccharides (FOS and GOS) either by enzymatic synthesis or by hydrolysis.
• The Life Cycle Inventory data and Life Cycle Impact Assessment data of this dataset are important to ensure more transparency in the LCA modeling involving FOS and/or GOS.
• These data can be beneficial to scientists and/or FOS/GOS producers and users who are interested in environmental impacts of such compounds.
• These data can be used as relevant information for assessment of food and biotechnology sector sustainability.
• These data can be used to suggest eco-design recommendations for more environmentally friendly food and biobased products production.

## 1 Data Description

All of the inventory data (LCI) of the oligosaccharides (FOS and GOS) produced either by enzymatic synthesis or substrate hydrolysis and the results of their environmental impacts (LCIA) are available in the associated dataset. An analysis of these data has been presented in a proceeding paper of LCA Food 2020 conference [1].

The dataset contains 9 files

1. Equipment_data: Data related to the equipment used, including power of the equipment, composition of the equipment (materials used and mass of each material).
2. FOS_HYD_LCI: Inventory data for all steps of the FOS production by hydrolysis of yacon, calculated for a FU of 100 g of oligosaccharide powder. The inventory includes all the flows taken into account, arranged by production steps. It is indicated whether the flow is incoming or outgoing, its quantity and the database and background data used to model the flow.
3. FOS_HYD_LCIA: Life Cycle Impact Assessment of the production of 100 g of powder of FOS by hydrolysis of yacon, calculated using the characterization method “EF Method (adapted) V2.00 / Global (2010)/with tox categories”.
4. FOS_SYNT_LCI: Inventory data for all steps of the FOS production by synthesis from sucrose, calculated for a FU of 100 g of oligosaccharide powder. The inventory includes all the flows taken into account, arranged by production steps. It is indicated whether the flow is incoming or outgoing, its quantity and the database and background data used to model the flow.
5. FOS_SYNT_LCIA: Life Cycle Impact Assessment of the production of 100 g of powder of FOS by synthesis from sucrose, calculated using the characterization method “EF Method (adapted) V2.00 / Global (2010)/with tox categories”.
6. GOS_HYD_LCI: Inventory data for all steps of the GOS production by hydrolysis of chickpeas, calculated for a FU of 100 g of oligosaccharide powder. The inventory includes all the flows taken into account, arranged by production steps. It is indicated whether the flow is incoming or outgoing, its quantity and the database and background data used to model the flow.
7. GOS_HYD_LCIA: Life Cycle Impact Assessment of the production of 100 g of powder of GOS by hydrolysis of chickpeas, calculated using the characterization method “EF Method (adapted) V2.00 / Global (2010)/with tox categories”.
8. GOS_SYNT_LCI: Inventory data for all steps of the GOS production by synthesis from lactose, calculated for a FU of 100 g of oligosaccharide powder. The inventory includes all the flows taken into account, arranged by production steps. It is indicated whether the flow is incoming or outgoing, its quantity and the database and background data used to model the flow.
9. GOS_SYNT_LCIA: Life Cycle Impact Assessment of the production of 100 g of powder of GOS by synthesis from lactose, calculated using the characterization method “EF Method (adapted) V2.00 / Global (2010)/with tox categories”.

## 2 Experimental Design, Materials and Methods

For this study, we followed the steps of the standard LCA methodology [2].

### 2.1 Goal and Scope

The aim of this LCA was to assess the environmental performance of two different production processes from different substrates as shown on Table 1.

**Table 1:** Production scenarios presented in this paper and substrate used in each production scenario.

Oligosaccharide	Production scenario	Substrate
FOS	Enzymatic	Sucrose
	Hydrolysis	Yacon potato
GOS	Enzymatic	Lactose
	Hydrolysis	Chickpea seeds

For this purpose, LCA were performed using data collected at the laboratory scale, supplemented with data from databases.

A cradle-to-gate approach was considered in enzymatic and hydrolysis scenarios under assessment that is, considering the extraction or substrate preparation to produce the required inputs and the production of FOS/GOS but not the phases of use and/or final disposal of FOS/GOS. This perspective was assumed since the production systems are at laboratory scale and the products through these productions schemes are not available in the markets yet. Among the processes considered throughout the production life cycle of both, centrifugation, purification, freezing and freeze-drying were performed after the extraction phase.

The LCA functional unit must be selected carefully to allow comparisons between the systems under study. Thus, the functional unit was defined as 100 g of FOS/GOS produced by enzymatic or hydrolysis synthesis.

In this LCA, no allocation procedure was required.

### 2.2 Description of FOS and GOS Production Scenarios

#### 2.2.1 Scenario 1 - Enzymatic Synthesis

**Fig. 1:**
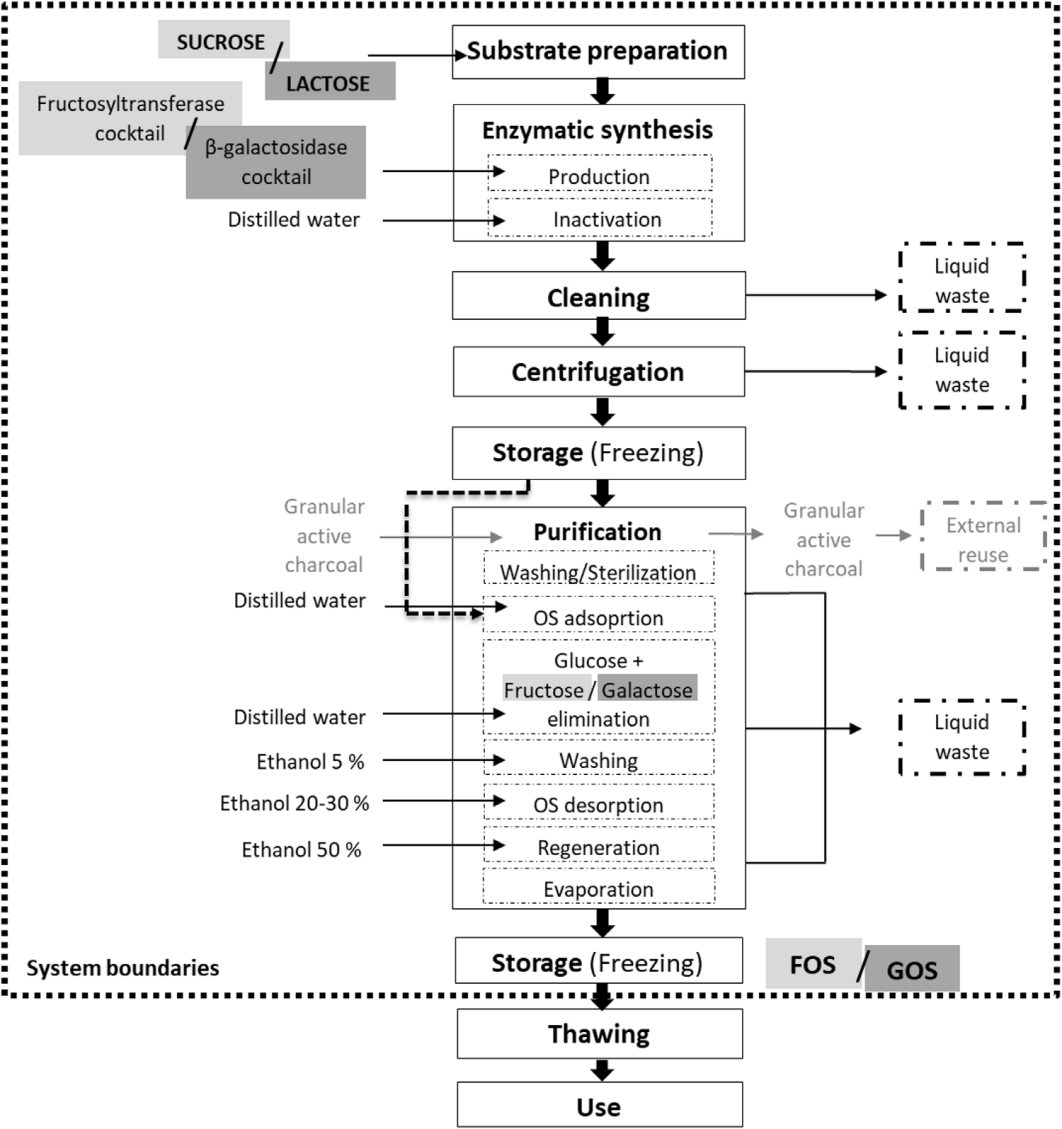
System boundaries and process chain under study corresponding to the enzymatic synthesis of FOS (light grey) and GOS (dark grey) production**.** Dashed lines indicate the system boundaries.

This scenario based on the enzymatic hydrolysis of sucrose and lactose was performed by fructosyltransferase and β-galactosidase enzyme cocktails, respectively [3]. The enzymatic production of FOS and GOS was composed of 7 mains steps which are depicted in Fig. 1: substrate preparation, enzymatic synthesis [production and inactivation], centrifugation, cleaning, storage, purification [including washing/sterilization, FOS/GOS adsorption, glucose/galactose elimination, washing, FOS/GOS desorption, regeneration, evaporation] and a last storage [freezing]. The system was assessed from the preparation of raw materials (resources) up to the final product at the laboratory scale. The transport activities, thawing and use of FOS/GOS were excluded from the system boundaries. The detailed description of FOS and GOS production by enzymatic synthesis is included below.

##### 2.2.1.1 Substrate Preparation

**(i) FOS Production:** Sucrose solution at 60% prepared in distilled water was used as substrate.

**(ii) GOS Production:** Lactose solution at 40% prepared in distilled water was used as substrate.

The pH of sucrose and lactose solution was adjusted to 5.5 by adding NaOH and then the solution was heated to 50 °C for 30 min in a microwave oven and then in a water bath used to maintain a constant temperature. In both situations (FOS and GOS), the final volume of the reaction medium was 0.960 L.

##### 2.2.1.2 Enzymatic Synthesis

The aim of this step was to produce mixtures of sugars as rich as possible of DP3 to DP5 compounds from dissacharides (DP2). The equipment used was a beaker (useful volume: 1 L) into which the reaction medium was poured and an enzyme cocktail was added at a concentration of 4% w/v. The final volume was 1 L. The operation was carried out for several hours at 50 °C with a agitation of 100 rpm.

- The enzyme cocktail used to produce FOS was Viscozyme L (56 FU/ mL; FU: fructosyltransferase units) according to [3];
- The enzyme cocktail used to produce GOS was Biolactase NTL (500 FU/ mL; FU: β-galactosidase).

After this phase, the syrup was diluted in half with distilled water and heated to 95 °C for 40 min, to denaturate the enzyme and thus stop the reaction.

##### 2.2.1.3 Centrifugation

To separate the enzyme from the rest of the syrup, a centrifugation step (12,000 g, 20 min at 4 °C) was applied.

##### 2.2.1.4 Cleaning

The cleaning solution was composed of a mixture of NaHCO3 (1% w/w) combined with H_3_PO_4_ (1% w/w).

##### 2.2.1.5 Storage

The FOS/GOS syrups were stored at −20 °C to avoid microbial contamination.

##### 2.2.1.6 Purification

The objective of this step was to remove monosaccharides from mixtures of sugars produced.

To this aim, activated carbon (charcoal) loaded in a chromatographic column was used.

###### 2.2.1.6.1 Washing/Sterilization

The first phase consisted of washing the column (2 L volume) with distilled water to remove fine particles. In the same way, the charcoal must be cleaned in a beaker. Once the water has been removed (charged with fine particles), the operation can be repeated several times. The charcoal used as a selective absorbent of oligosaccharides (0.750 kg) was a granular type [0.5–1.0 mm 18–35 ASTM (La Plata, Argentina)].

Before charging the column with charcoal, it is important to remove air bubbles from the pores of the charcoal. The following step was to redispose it in a beaker filled with distilled water (about 2.5 cm above the charcoal level) to sterilize it at 121 °C for 15 min (autoclave).

The charcoal was then loaded with a spatula into a column filled with distilled water. A final cleaning was carried out (6 L of distilled water) to remove the remaining fine particles. A flow of 20 mL/min in the peristaltic pump was applied.

###### 2.2.1.6.2 Adsorption and Elimination

To selectively adsorb oligosaccharides, 1 L of syrup (FOS/GOS) at 30% (w/v) was treated by recirculating water at 30 mL/min flow for 1 h. At the end of the process, 1 L of syrup was collected containing the unabsorbed fraction.

A cleaning step of the column was carried out for removing mono and disaccharides by recirculating 2 L of distilled water at 30 mL/min. The water loaded with mono and disaccharides used was discarded. The unabsorbed fraction previously obtained (1 L) was recirculated again through the charcoal at 30 mL/min flow for 1 h. The unsorbed fraction was collected and washed. This protocol of adsorption and elimination of mono and disaccharides was repeated three times to promote the adsorption of oligosaccharides on charcoal. Throughout this stage, the syrup was kept at a temperature of 40 °C.

###### 2.2.1.6.3 Washing

In order to improve the elution of monosaccharides and disaccharides still adsorbed by charcoal and to increase the purity of FOS, a washing step was applied. First, the column was washed by circulating 4 L of distilled water and 2 L of 5% v/v ethanol. During the last washing step, the sugar composition (% w/w) was mainly enriched with mono and disaccharides. This final washing phase was repeated until the concentration of monosaccharides (FOS: glucose and fructose; GOS: glucose and galactose) reached a minimum.

###### 2.2.1.6.4 Desorption/Regeneration

Sugars (DP1 to DP5) were selectively adsorbed on activated charcoal. FOS were adsorbed more strongly than mono- and disaccharides. Thus, an ethanol gradient with an increasing concentration from 20 to 50% (v/v) was used to desorb the FOS/GOS: 2.5 L of 20% v/v ethanol, 2.5 L of 30% v/v ethanol and 1 L of 50% v/v ethanol. During desorption, 0.5 L aliquots for each ethanol concentration were collected. The desorption step was carried out slowly (select 15 to 20 mL/min of flow in the peristaltic pump).

###### 2.2.1.6.5 Evaporation

The fractions collected during the desorption phase were evaporated to remove the ethanol and concentrate the sugars prior to freeze-drying. The ethanol was evaporated at 60 °C with a rotary evaporator (Büchi Rotavapor, Flawil, Switzerland) (useful volume: 3 L). The quantity obtained was 100 g of FOS/GOS powder.

##### 2.2.1.7 Storage

The oligosaccharides produced were stored at −20 °C in a laboratory freezer (300 L) until using them.

#### 2.2.2 Scenario 2 – Hydrolysis Synthesis

**Fig. 2:**
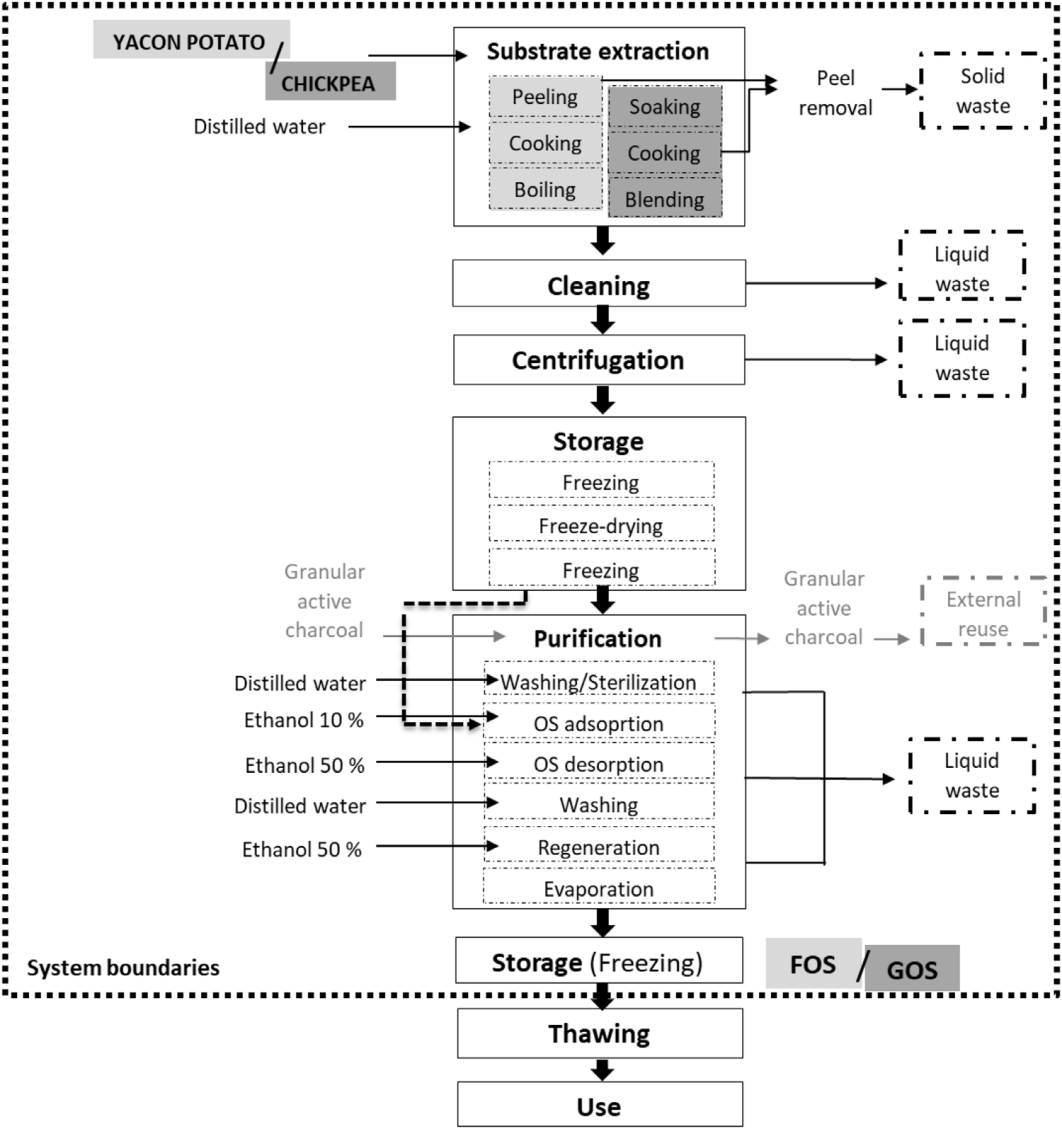
System boundaries and process chain under study corresponding to the hydrolysis synthesis of FOS (light grey) and GOS (dark grey) production**.** Dashed lines indicate the system boundaries.

This autohydrolysis of yacon potato and chickpea seeds is done by boiling the cleaned and cut or ground up tubers, or other inulin containing plant part, in water [4]. The production of FOS and GOS by hydrolysis was divided into 6 main steps which are depicted in Fig. 2 (**a** and **b**): substrate extraction [matrix preparation/soaking, extraction/cooking and concentration], centrifugation, cleaning, storage [freezing, freeze-drying and freezing] and purification [washing/sterilization, FOS/GOS adsorption, FOS/GOS desorption, washing, regeneration, evaporation] and a last storage [freezing]. The system was assessed from the preparation of raw materials (resources) up to the final product at the laboratory scale. The transport activities, thawing and use of FOS/GOS were excluded from the system boundaries.

The substrate extraction method used to produce FOS was different from the one used for GOS.

##### 2.2.2.1 Substrate Extraction

**(i**) FOS production: Yacon potato (vegetable belonging to the Asteraceae family) is known for its high concentration of FOS. To obtain 100 g of FOS powder, 5.250 kg of yacon was required (proportion of peeling: 5% (w/w)). The material was heated with a hot plate to 75 °C for 60 min in 3 L of distilled water. The second phase consisted of boiling the preparation for 1 h in 2 L of distilled water without stirring.

(ii) GOS production: 5 kg of peeled chickpea seeds (family Fabaceae) were used (proportion of peeling 5% (w/w)) to obtain 100 g of GOS powder. The chickpeas were soaked overnight (about 12 h) in 3 L of distilled water and cooked using a pressure cooker at 100 °C for 30 min.

The preparation was suspended in 2 L of distilled water and then, incorporated in a blender (5 min, 50 °C). The purpose of this step was to homogenize the preparation of chickpeas in distilled water.

##### 2.2.2.2 Centrifugation

A centrifugation was carried out (12,000 g, 10 min at 4 °C) to eliminate the organic matter (4 kg of organic waste) from the rest of the preparation.

##### 2.2.2.3 Cleaning

The cleaning solution was the same as that used for the cleaning of the FOS/GOS enzymatic synthesis production system.

##### 2.2.2.4 Storage

The supernatant obtained from centrifugation was frozen at −20 °C and freeze-dried in laboratory type equipment (Heto FD4, Heto Lab Equipment, Denmark). The freeze-dried sample was stored (approximately 1 week) in a full-scale laboratory freezer (300 L).

##### 2.2.2.5 Purification

The phases performed in this step production were similar to those described for the enzymatic synthesis production system (Section 2.2.1.6), except for the ethanol concentration, described in Table 2.

**Table 2:** Ethanol concentration used for purification in the hydrolysis process.

Step	Ethanol concentration (% v/v)
Adsorption	10
Desorption	50
Regeneration	50

##### 2.2.2.6 Storage

The final product was stored at −20 °C.

### 2.3 Inventory Data

Inventory data for the foreground system (direct inputs and outputs for each stage) have been collected during experiments.

#### 2.3.1 Equipment

Different equipment have been used for the experiments: microwave (BGA, Argentina), fermenter, heating plate (IKA HS 7, USA), centrifuge (Beckman Coulter Inc., USA), freezer (PEETLAB – MO398S / MO528S, USA), autoclave (Chamberlain VZ-100, Argentina), pump (Gilson, Middleton, WI, USA), chromatography column (Benson Polymeric, Reno, NV, USA), rotavapor (Büchi Rotavapor, Flawil, Switzerland), pressure cooker (MARMICOC, Argentina), freeze-dryer (Heto Lab Equipment, Denmark). The mass and nature of material they are composed of have been obtained from equipment user manuals or manual measurement when possible and considered in LCI with a temporal allocation (Eq. (1)). The lifetime of the equipment has been considered to be 30 years.

(1) $$Temporal\;allocation\;factor=\frac{Duration\;of\;use\;of\;the\;equipment\;\left(h\right)}{Lifetime\;of\;the\;equipment\;\left(h\right)}$$

The equipment data are provided in the Equipment–data file and the resulting inventory data related to equipment are available in LCI files in the dataset.

#### 2.3.2 Mass Flows

Mass flows of ingredients, water, cleaning products, product losses and wastes have been either manually measured or estimated during the experiments.

Loss of refrigerant of freezer has been calculated as in [5] (Eq. (2)).

(2) Lossofrefrigerant=Equipmentpower*Refrigerantcharge*Leakagerate

The power of the freezer was noted on the identification plate directly on the freezer. The refrigerant charge was obtained from user manual. The annual leakage rate of refrigerant contained in the cold room was assumed to be 15% [6,7].

The resulting inventory data related to mass flows are available in LCI files in the dataset.

#### 2.3.3 Energy Flows

Electrical consumptions (kWh) have been calculated from equipment powers *P* (kW) and their duration of use *t* (h) (Eq. (3)).

(3) Electricalconsumption=P*t

The power of each equipment was noted on the identification plate directly on the equipment. The equipment powers are provided in the Equipment_data file and the resulting inventory data related to electrical consumptions are available in LCI files in the dataset.

#### 2.3.4 Background Data

The inventory data corresponding to the production of the different inputs to the systems (ingredients, electricity, alcohol, tap water) and the wastewater treatment process were taken from Ecoinvent database v3.5 by using the cut off system model. The laboratory scale process was located at CIDCA-CONICET (La Plata, Argentina) and University of Madeira (Madeira, Portugal), so the average electricity generation and imports/exports from Argentina and Portugal have been considered as GLOBAL in terms of geographical precision in the database. Yacon potato could not be found in any database and was approximated with Banana production process. Lactose was also approximated, with cow milk production process.

### 2.4 Impact Characterization

SimaPro (v9.0.0.35 Pre consultant) was used for the impact characterization with the EF Method (adapted) V2.00 / Global (2010)/with tox categories. All the midpoint impact indicators available in this method have been calculated: Climate change, Ozone depletion, Ionising radiation, Photochemical ozone formation, Respiratory inorganics, Non-cancer human health effects, Cancer human health effects, Acidification terrestrial and freshwater, Eutrophication freshwater, Eutrophication marine, Eutrophication terrestrial, Ecotoxicity freshwater, Land use, Water scarcity, Resource use - energy carriers, Resource use - mineral and metals, Climate change – fossil, Climate change – biogenic, Climate change - land use and transform.

## Ethics Statements

This work did not involve human subjects or laboratory animals, and therefore did not encounter any ethical issues.

## CRediT authorship contribution statement

**Esteban Gerbino:** Investigation, Resources, Writing – original draft. **Camille Quentier:** Methodology, Investigation, Data curation, Writing – review & editing. **Caroline Pénicaud:** Conceptualization, Methodology, Writing – review & editing, Supervision, Project administration, Funding acquisition.

## Declaration of Competing Interest

The authors declare that they have no known competing financial interests or personal relationships that could have appeared to influence the work reported in this paper.

## Data Availability

Life Cycle Assessment of fructo- and galacto-oligosaccharides (FOS and GOS) produced by synthesis or hydrolysis (Original data) (Dataverse).
